# Prelnc2: A prediction tool for lncRNAs with enhanced multi-level features of RNAs

**DOI:** 10.1371/journal.pone.0286377

**Published:** 2023-06-01

**Authors:** Hua Gao, Peng Gao, Ning Ye

**Affiliations:** 1 College of Forestry, Nanjing Forestry University, Nanjing, China; 2 College of Information Science and Technology, Nanjing Forestry University, Nanjing, China; 3 The First Affiliated Hospital of Xi’an Jiaotong University, Xi’an, China; Nuclear Science and Technology Research Institute, ISLAMIC REPUBLIC OF IRAN

## Abstract

Long non-coding RNAs (lncRNAs) have been widely studied for their important biological significance. In general, we need to distinguish them from protein coding RNAs (pcRNAs) with similar functions. Based on various strategies, algorithms and tools have been designed and developed to train and validate such classification capabilities. However, many of them lack certain scalability, versatility, and rely heavily on genome annotation. In this paper, we design a convenient and biologically meaningful classification tool "Prelnc2" using multi-scale position and frequency information of wavelet transform spectrum and generalizes the frequency statistics method. Finally, we used the extracted features and auxiliary features together to train the model and verify it with test data. PreLnc2 achieved 93.2% accuracy for animal and plant transcripts, outperforming PreLnc by 2.1% improvement and our method provides an effective alternative to the prediction of lncRNAs.

## Introduction

Long non-coding RNAs (lncRNAs) are a type of RNA with a limited capability of encoding proteins, whose lengths are usually over 200 bp [1]. lncRNAs play a role in regulating gene expression in cells, thus lncRNAs are often used for life regulation research and targeted drug research [2]. However, the roles of lncRNAs are not yet fully studied, partially due to the incomplete annotation of lncRNAs [3]. Therefore, there is an urgent need to distinguish lncRNAs from other types of RNAs in the transcriptome.

However, designing efficient algorithms to accurately predict lncRNAs is highly non-trivial due to three main reasons. First, the similarities of the structural and biological functions between lncRNA and pcRNA [4] make it mathematically and biologically easy to misclassify lncRNA as others. Moreover, some high-abundance lncRNAs are poorly conserved, but some low-expression lncRNAs have crucial functions [5–9]. Such differences in abundance directly affect the balance of sample classes. Therefore, it is not easy to directly learn a certain non-coding capacity from lncRNAs with different expression abundances, different degrees of conservation, and different species.

Even though it is difficult to predict lncRNAs, great efforts have been devoted to this issue with various strategies. Existing methods fall into two main categories. One is to experimentally verify the protein-coding ability of RNA, which is often costly and time-consuming, but the accuracy of the results is very high. This approach is commonly performed only for identification and functional verification of RNAs of interest. Another approach is to extract some features from the existing sequence data by exploiting statistical information or some annotated information of the sequence to characterize the coding ability of the sequence. This approach is relatively economical, less time-consuming, and can be carried out in large quantities. In addition, the accuracy is relatively lower compared to the experimental methods. Currently, this approach is mainly used to screen a large number of possible lncRNAs. Specific biological experiments are subsequently performed to verify and study its function. As a result, most researchers prefer to use these computational methods to conduct their research first.

Current algorithms can be roughly classified into two groups based on their strategies for exploiting annotated data. One is to use known biological annotation databases to perform homology blast to find possible coding information. They are experimental and annotation database-dependent methods, such as using the structural characteristics of RNA sequences to predict the coding ability of RNA. PhyloCSF, for example, uses sequence evolution features to determine whether it is a lncRNA or not. Some tools use protein profile data, ribosome analysis, Swiss Prot or UniProt knowledge database comparison, etc. [10, 11]. While these methods rely on annotated information to achieve some accuracy, they are generally only effective for sequences with a lot of studied information and lack resolution for recently studied or unstudied species. More typically, this approach of continually querying remote databases is inefficient and costly when there is a large amount of data.

The other group is to obtain features by directly computing or aggregating statistics from transcriptome sequences, some of which are carefully designed or not, and then using classical classifiers to identify it, that is, sequence-computed dependency types. There are currently two main directions for this group. One uses the designed features for classification directly, such as CPAT [12], PLEK [13], CPC2 [14], lncRScan SVM [15], LncFinder [16] and Prelnc [17]. CPAT is a tool for evaluating the coding ability of proteins based on linear regression models. CPC2 uses base ORF features, Fickett Score and PI, PLEK use K-mers and then feed SVM with features for classifying. LncFinder utilizes sequence intrinsic composition, structural information, and physicochemical properties to identify lncRNAs. These features mainly depend on the manual discovery and statistical analysis of the features, so there is an urgent need for a way to unearth the vast amount of hidden information, and thus the machine learning methods based on hidden information have emerged.

The successful application of deep neural networks in other fields provides a lot of possibilities for biological data mining [18]. Neural network-based lncRNAs classifiers, especially deep neural networks, have emerged at the right moment. Typical tools are LncADeep [19] and DeepLNC [20]. LncADeep integrates intrinsic and homological features into a deep belief network and constructs models that target both full-length and partial-length transcripts. Obviously, this is very unfriendly to species that are originally studied and/or have a little annotated data. Unlike LncADeep, DeepLNC uses K-mers pattern features directly calculated from sequences to feed the network for training and classification. They are effective for prediction, but a critical challenge lies in understanding which meaningful features a network is actually learning.

As a result, most researchers prefer tools that can directly compute features with significant biological interpretation. It is also up to these features that can guide drug and breeding research. However, existing lncRNAs prediction tools still suffer from some problems. For example, the number of targeted species on which can be applied is scarce, particularly for plants. In addition, it is difficult for plant prediction tools to obtain high-accuracy predictions on plant lncRNAs data, which are somewhat biased in terms of positive and negative values due to the large number of pcRNAs and the limited number of lncRNAs. This issue directly leads to the difficulty of efficiently improving the accuracy of classifiers based on features obtained by statistical methods [21]. In order to effectively improve the accuracy, some researchers try to obtain signal features by signal processing. LncFinder, for example, they first select a known amplitude conversion matrix to transform biological sequences into signal sequences, and then use some mature signal processing tools to analyze the physical signal information characteristics of the sequence and to extract them.

The two main signal analysis tools are Fourier transform and wavelet transform. The former can extract the frequency information of biological sequences, but it is difficult to give the statistical properties of biological sequences at different sites, which is similar to the K-mers method. Although STFT (short time Fourier transform) was used later, the classification effect is still poor due to its window effects. The wavelet transform was developed to overcome the shortcomings of the Fourier transform, but there are some obvious drawbacks in applying it to biological sequential signals. For example, the current approach is simply to extract the peak of the site or target site of the drug from the angle of the signal. In other words, it is difficult to design features that give energy distributions at different locations in biological sequences of different lengths. Furthermore, this signal processing approach does not or cannot lead to a more biologically significant and understandable approach to extract features from RNA sequences. Therefore, we propose a wavelet spectral-based feature extraction method to extract location distribution features in the energy spectrum, and then used these features to assist existing literature features to improve the classification effect of plant lncRNAs.

## Materials and methods

### Datasets

There are mainly three datasets in this paper, the first dataset, major for training and testing the model, can be divided into two categories, one consisting of 3 animals (*Human*, *Mouse* and *Cow*), the other one consisting of 3 plants (*Arabidopsis thaliana*, *Oryza sativa*, and *Zea mays*), this dataset is available online at https://github.com/LeiCao97/PreLncData, detailed information of the dataset is described in [17]. In order to effectively verify the effectiveness and generalization ability of the program, we introduced three datasets, the CPC2 dataset [22] and NoNcodeV6 [23], NONcodeV5 [24]. Part data of CPC2 and NoNcodeV6 are used to verify the generalization prediction ability of uncalibrated data, because they cannot be directly verified and compared with other software.

### Feature selection and extraction

**Fig 1:**
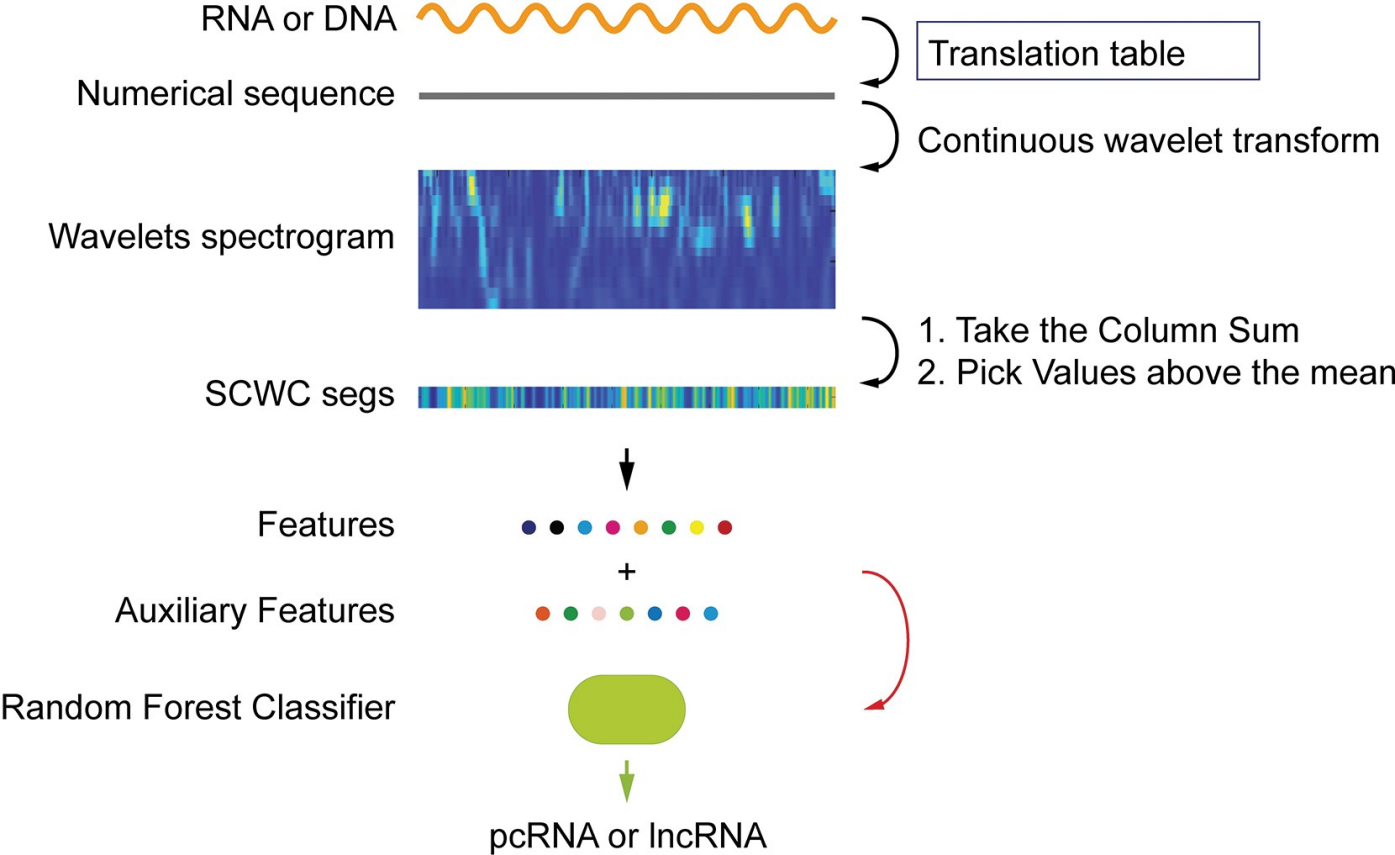
The flow of wavelet feature extraction. An RNA or DNA sequence is first transformed into a numerical sequence by the translation table, and then a wavelet transform is applied and the result scalogram will be summed by columns. some features are extracted according to certain rules, and finally, all the features are filtered for relevance before being fed to the classifier.

This section will introduce the workflow as shown in Fig 1, including transforming RNA into the numerical sequence, extracting wavelet scalogram and extracting features, and finally removing some features according to correlation, and finally feeding them into random forest classifier for classification.

#### The first subset of 8 features

To make the model more accurate and more generalized, we choose some features that have the following properties: First, it can be computed directly by the program. Second, it can effectively enhance the accuracy and precision of predictive model. Thirdly, it has been widely used by literatures in recent years. Finally, we select eight auxiliary features based on their degree of relevance (Table 1).

**Table 1:** The first subset of eight features.

Features Categories	Feature	Introduction
Basic features	seq_len	Sequence length
	GC_content	GC content
	Stop_std	Standard deviation of stop codon counts (TAA, TAG, TGA)
ORF related	Orf_fullness	Open reading frame integrity
	CDS_Score	CDS Score of txCdsPredict prediction
	CDS_percent	CDS percentage
Peptide related	PI	Isoelectric point
Functional definition related	Fickett_Score	Fickett Score

The first basic set of features are numerical statistical features that can be obtained directly from sequence data [25]. The structural characteristics of the ORF (open reading frame), including ORF integrity, CDS Score, and CDS percentage are crucial features [15, 26, 27]. To some extent, these three features play a decisive role in the discrimination of lncRNAs. In particular, in most of the literature, the CDS Score contributes more than 50%~75% to the accuracy. Physical feature theoretical isoelectric point (PI) [16] which provides some statistics of the number of acidic and alkaline amino acids in peptide chains is used for many predicting software. These features can be directly calculated by python packages biopython [28]. Fickett Score is described in the literature [12, 29]. The first set of features can only provide 67.6% to 88.3% accuracy when classified individually on the datasets in this paper, so the second set is needed to further improve the accuracy.

#### The second subset of wavelet multi-scale features

The way sequences are treated as signals facilitates the discovery of certain biochemical properties with well-established signal analysis tools. The first fundamental problem is how to transform the sequence into a suitable signal amplitude. Feature PI [28] uses a translation table of pH properties of nucleotides to convert a nucleotide sequence to a numeral signal sequence and then perform Fourier transform to extract features. Until now, there have been many methods to transform sequences into numerical values, and they all have specific research backgrounds. Here, we adopt the same transformation method as in PI.

The second problem is how to choose the right signal analysis tool. In general, scholars tend to use the K-mers to design certain frequency features of biological sequences, and the corresponding method in signal processing is the Fourier transform. However, it is not easy to gain regular characteristics from non-stationary biological sequences, so many analysis methods are carried out using modern tools such as STFT or wavelet transform. In this work, we adopt the tool of wavelet analysis for feature extraction from RNA sequences. For one hand, Wavelet transform is a relatively mature signal analysis tool, and It can directly find the charged properties of non-stationary biological sequences [30]. and on the other hand, the wavelet transform can also give some charged properties of RNA sequences at different scales and positions. This is an advantage over the Fourier transform and STFT methods, which only look for the frequency statistical properties of the charged properties.

In addition to the two obvious advantages above, we used Wavelet analysis based on such an assumption: If the transcriptome is regarded as a non-stationary charged signal chain, its biological characteristics suggest that it has such a possible property that coding RNA is more inclined to encode rather than adsorb on other chains to regulate transcription or cleavage. In other words, lncRNAs are more inclined to attract or attack other chains or proteins, thereby increasing the chance to achieve the purpose of modification and regulation. It is possible that certain regions of lncRNAs chains exhibit much more efficient charged adsorption than those of pcRNAs chains, but we know that such adsorption is also present in general coding RNA, for example, in RNA involved in mediation. Thus, based on such considerations, wavelet analysis can improve on the shortcomings of Fourier’s inability to provide feature information for position-based modification capabilities, and some results can be obtained by wavelet spectral analysis regardless of the hot spot location, length, and repeated patterns or charge patterns on different spans. Therefore, it is feasible to extract the features of each sequence by analyzing the wavelet spectrum.

**Fig 2:**
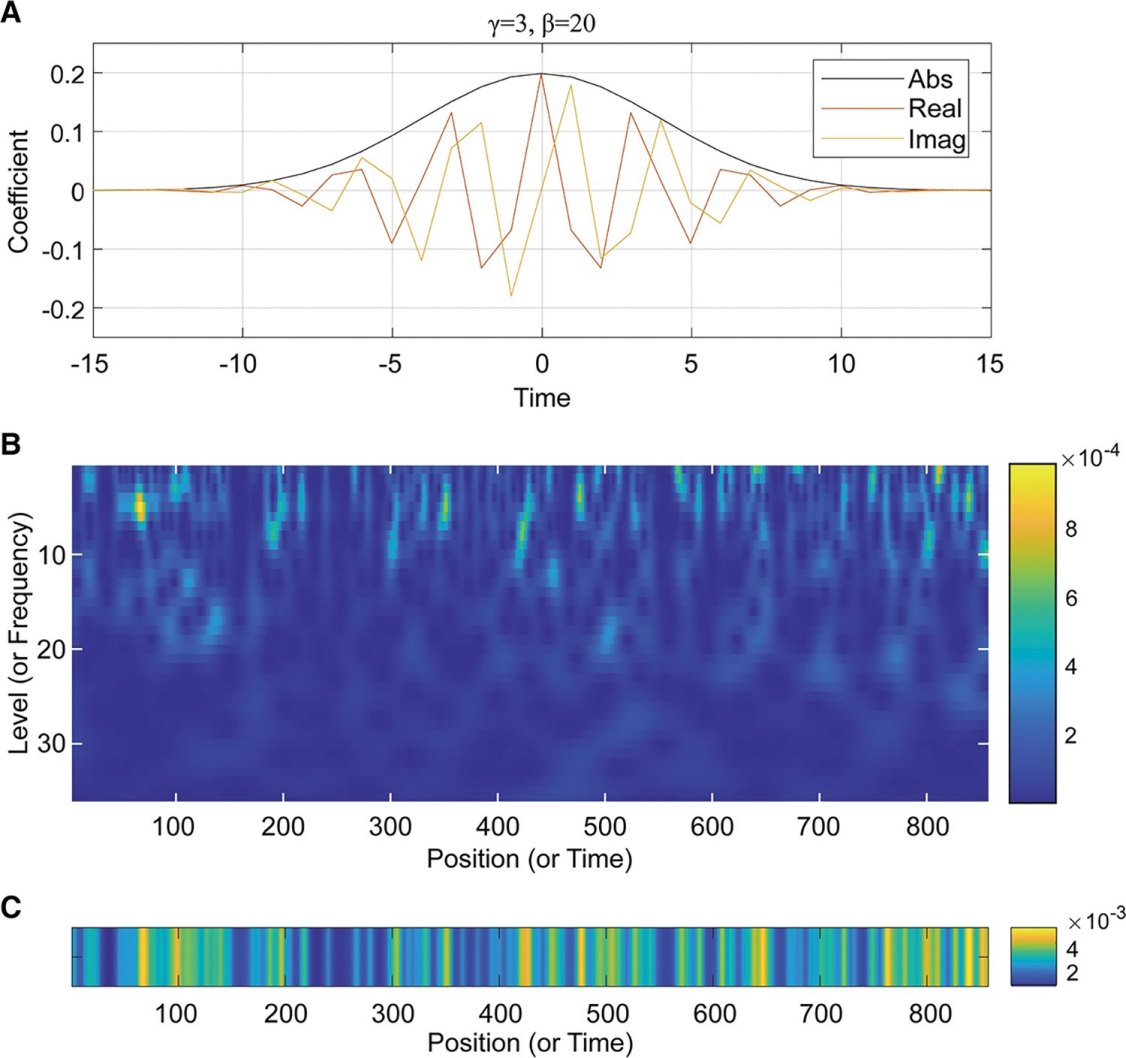
Morse wave shapes, time-frequency scalogram and sum of wavelet transform map. (**A**) Morse wave shapes, the black line shows the normal distribution, and the remaining-colored lines show their imaginary and real components. (**B**) Wavelet transform map of an RNA sequence, The highlighted parts of the scalogram show that it has some high electrostatic properties, and the y-axis shows the characteristics at different levels. (**C**) The column sum of the time spectrum.


In order to show the peak features of the sequential signal rather than highlight the selected frequency features, we choose waveforms with a Gaussian distribution. In this work, the Morse wavelet is selected (formula is Eq (1), Morse wave is shown in Fig 2A [31]), and the Morlet wavelet can also achieve the same effect. Here we used the default parameters in many literatures, that is, β=20,γ=3 [32]. For example, based on this wavelet, we converted a sequence of more than 800 bp into a wavelet time spectrum (Fig 2B).

$$\varphi (\beta ,\gamma ,\omega )=U(\omega )a_{\beta ,\gamma }\omega^{\beta }e^{-\omega^{\gamma }}$$ (1)

Fig 2B shows the time-frequency scalogram of an RNA sequence after wavelet transformation (1–35 layers wavelet coefficient energy). The y-axis represents the wavelet coefficients at different scales (or frequencies), that is, the charge potential fits to the Gaussian peaks at different scales. The brighter the image, the higher the fitness of the charge capacity potential, which typically decreases at about 10–20 layers, indicating that this potential is typically displayed up to 10–20 base lengths. In other words, the sequence of Gaussian waveforms fitting the features between 1 and 20 is relatively visible.

At the same time, the lengths of the various luminosities along the x-axis show a characteristic frequency at a particular length, and this point in the small spectrum is very similar to K-mers. There is, however, something more important than length, the x-axis directly provides information about the location of this expected K-mers. Given such a clear phenomenon, we further hypothesize a process in which the regulatory target is first broadly attracted by long-high potential sites, and then small-low potential sites are precisely matched to complete the regulation. Therefore, the generalized attraction is the first step, and the exact matching is the next. Thus, our approach is to look for such patterns as a possible first step to train useful features for classification.

**Fig 3:**
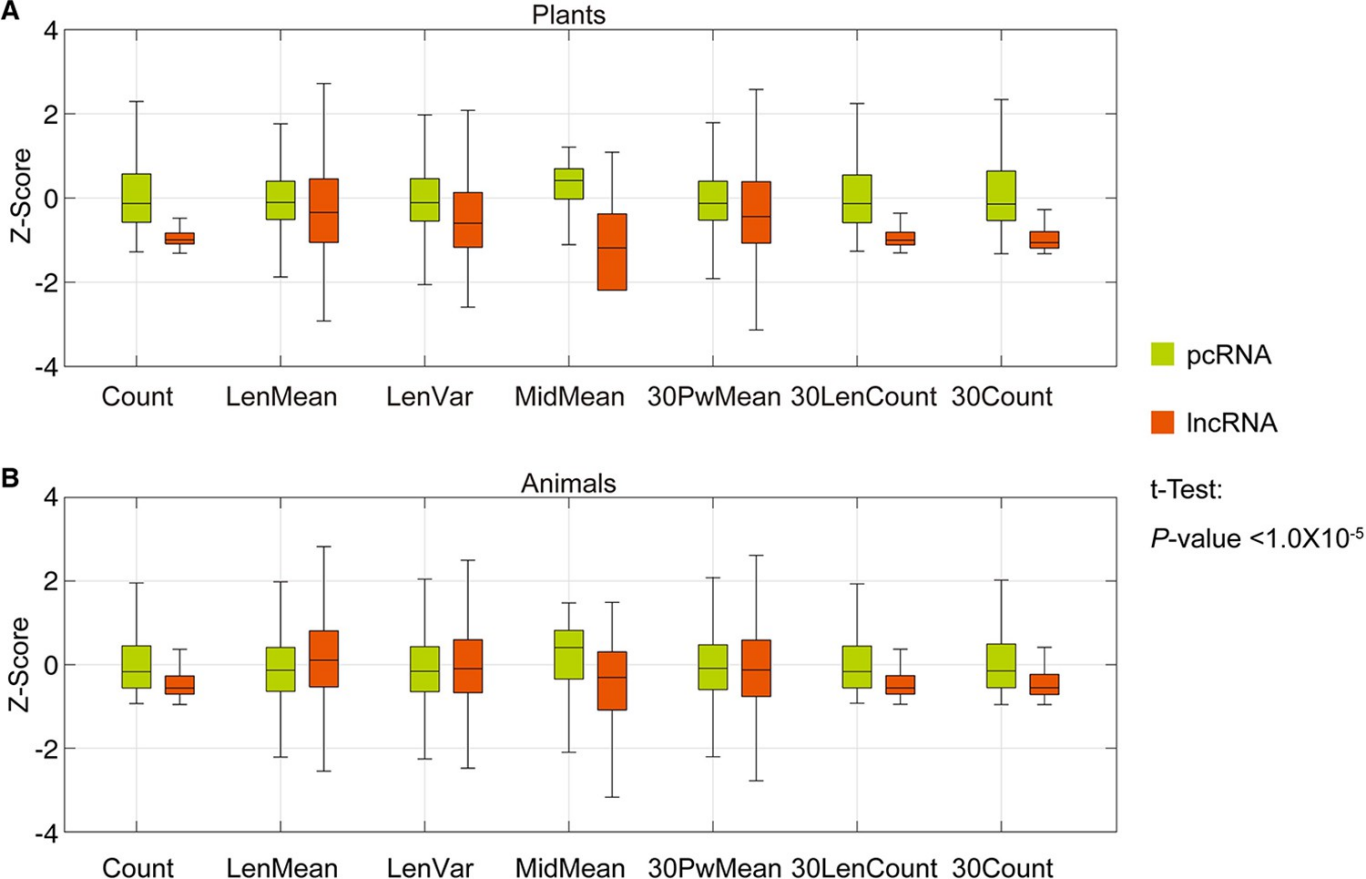
The wavelet characteristic distribution by groups. (**A**) Z-Score distribution of the 7 Wavelets features in plants. (**B**) Z-Score distribution of the 7 Wavelets features in animals. The t-test hypothesis test shows that they are all significantly different, with a *P*-value significantly smaller than $1.0\times 10^{-5}$.

In order to extract the position and frequency information in the transform spectrum, we adopt such a method (Fig 2C). We first transform the transcriptome sequence into the corresponding numerical sequence. The second step is to transform the numerical sequence into the corresponding wavelet transform spectrum using a Morse wavelet. We then sum the absolute values of the transformed spectra of layers 1–20 over the time and frequency spectra. The goal is to simplify the number of features by squeezing nearly 20 layers of features into a single generalized layer of latent features. We abbreviated the spectrum corresponding to the column sum of the wavelet transform matrix as (SCWS). The part of SCWS that is greater than the average value is taken (SCWSS), and we get such a spectrum, as shown in Fig 3. Finally, 7 different feature information (Table 2) are selected according to the sum of columns.

**Table 2:** Features extracted from wavelet transform.

No.	Feature	Introduction
1	Count	Total number of fragments above average power.
2	LenMean	Average of length of fragments.
3	LenVar	Variance of length of fragments.
4	MidMean	The fixed mean of the midpoint positions of segments.
5	30Count	The total number of fragments whose length are greater than 1/3 of the total number of sorted fragments and whose length is over 30.
6	30PwMean	Average power whose length is greater than 1/3 of the total number of sorted fragments and whose length is over 30.
7	30LenCount	Average length of fragments whose length is over 30.

The formula of the fixed mean of the midpoint positions of segments is Eq (2). This formula considers the problem of the inability to standardize one data when predicting a single lncRNA, so that this formula can easily control the midpoint value to a number less than 1.


$$FMid=\frac{1}{2}\frac{{P_{end}-P}_{start}}{P_{end}}$$ (2)

In designing wavelet features, in order to achieve the goal that the number of SCWSS cannot be highly correlated with the length of the RNA sequence and that it cannot be biologically irrelevant, two filtering conditions are given here: the fragments with a length greater than 30 (30-up) and the fragments are greater than 1/3 of the total number of sorted fragments. The first condition is the nucleotide length corresponding to the shortest known protein, so that some coding sequences that may not be meaningful can be excluded. The second condition is based on the consideration that a large number of high-energy regions with relatively short length in the scalogram within 1–6 layers will be directly displayed in SCWSS, while SCWSS within more than 6 layers are relatively long and few in number, and these 6 layers account for exactly 1/3 of the 20 layers. Features 30Count and 30PwMean are under these two common conditions, and 30LenCount is under the first condition. The statistical results show that compared with the animal, 30PwMean and 30Count in plants are easier to distinguish.

### Model evaluation parameters

The performance of the model in this paper was compared with mainstream and latest tools, including the best ones like LncFinder, Prelnc, and CPC2, refer to the paper [17], we evaluated the performance using standard methods including accuracy (ACC), area under curve (AUC), sensitivity (SEN), specificity (SPE), Matthews correlation coefficient (MCC), they were described by Eqs (3–7). The exact interpretation is given in ref [17].


$$ACC=\frac{TP+TN}{TP+TN+FP+FN}$$ (3)

$$AUC=\frac{\sum_{+}rank_{i}-0.5(M(1+M))}{M\times N}$$ (4)

$$SEN=\frac{TP}{TP+FN}$$ (5)

$$SPE=\frac{TN}{TN+FP}$$ (6)

$$MCC=\frac{TP\times TN-FP\times FN}{\sqrt{\left(TP+FP)\times (TP+FN)\times (TN+FP)\times (TN+FN\right)}}$$ (7)

## Results

### Analysis of feature selection

In this section, we first verify the classification ability of wavelet features for plants, and then combine wavelet features with auxiliary features. These combined features are tested on the dataset using a random forest classifier. We used the 5 indicators to compare the pros and cons of several tools, and finally verify its generalization ability on the cpc2 dataset.

#### Wavelet features of RNAs significantly discriminate lncRNAs and pcRNAs

In order to enable the features to be used for classification, we first need a good degree of discrimination for the selected features. Thus, we first consider how the features are distributed.

We extracted the corresponding features of RNA sequences from the animal and the plant which are mentioned in Wavelet multi-scale features and then used Z-Score for statistical mapping (as shown in Fig 3, which filters out abnormal data points). The results show that the number of continuous fragments which are higher than the mean value of the time-frequency spectrum after wavelet transform is significantly different between lncRNA and pcRNA, and this difference are more obvious between animals and plants. The distribution of the number of SCWSS in plants is not only significantly different from that of pcRNA, but also lower than that of animals. For example, SCWSS segments differ significantly in length and 30-up length, which may be due to bias in the plant data, or to the fact that SCWSS have more homologous and variable shear patterns in animals.

In fact, the length of the SCWSS and its variance also show more pronounced differences in plants. The distribution of SCWSS lengths for lncRNAs in Fig 3 is more concentrated and shorter. As a result, lncRNAs does not encode relatively well. Even if it can code, the encoded proteins are relatively short, and many of them have no practical meaning. In addition, the distribution of the mean values of the corrected midpoints of the SCWSS shows some differences between plants and animals. Compared to lncRNA, pcRNA is more concentrated and has relatively high values, indicating that the functional coding positions in pcRNA are relatively evenly distributed and stable. This may be due to the need to encode more stable features of RNA during evolution. However, lncRNA may be degraded or transposed from coding RNA, which has some coding capacity, but more adsorption capacity to other RNA or DNA. All of this suggests that the features extracted by the wavelet can build a relatively strong classifier for distinguishing lncRNA from pcRNA in plants.

#### Wavelet features significantly improve the accuracy of prediction of lncRNAs

We classify the datasets using only wavelet features and give the accuracy and AUC values; the results are shown in Table 3. The accuracy of the three animals in Table 3 is generally lower than that of the plants. The plants met the strong classifier criterion of 80 percent, while the animals met the weak classifier criterion at a relatively low level. This suggests that wavelet features alone can be used as a strong classifier on a given dataset, but the effect is not satisfactory as the subsequent AUC shows that it is not a very good strong classifier.

**Table 3:** Accuracy and AUC of wavelet feature classification.

Species	ACC (%)	AUC (%)
*Humans*	67.37	65.63
*Mice*	66.52	64.64
*Cows*	71.03	71.37
*A*. *thaliana*	81.78	78.70
*O*. *sativa*	90.09	84.73
*Z*. *mays*	85.83	85.24

#### Combination of features

To fully enhance the prediction accuracy and to build a better model, the two sets of features are combined and screened. A correlation Pearson correlation coefficient test was performed on the data, removing features with a correlation greater than 0.8, and finally selecting the same features for animals and plants. These 15 features have excellent predictability.

### Comparison and analysis of prediction result

We will compare three current tools (Prelnc, CPC2, and LncFinder) with relatively high accuracy. The results (Table 4) show that our tool is generally more accurate than other tools. The mouse data is slightly less precise, but the difference is negligible. Meanwhile, the performance of SEN and SPE again demonstrates that combinatorial features can considerably enhance the ability to classify RNA into lncRNA and pcRNA. It is likely that the poor performance of SEN on *O*. *sativa* is due to its pure species character, but this problem needs to be investigated further. So far, our tool has improved by an average of 2% over other tools and for plants it is 4.3% and achieved an average accuracy of 93.168%.

**Table 4:** Comparison of predicting results.

Species	Methods	SEN%	SPE%	ACC%	MCC%	AUC%
*Human*	Prelnc2	94.35	**87.95** ^a^	**89.44**	**75.05**	**96.85**
	Prelnc	**96.63**	85.00	87.70	72.80	96.20
	CPC2	94.75	69.40	75.29	54.40	90.13
	LncFinder	95.67	84.01	86.71	70.78	/^b^
*Mice*	Prelnc2	94.51	89.23	89.78	65.28	**97.27**
	Prelnc	94.16	**89.93**	**90.39**	66.53	97.09
	CPC2	94.79	76.24	78.26	47.69	97.17
	LncFinder	**95.65**	89.45	90.22	**66.56**	/
*Cow*	Prelnc2	**96.10**	94.00	94.06	51.95	**98.38**
	Prelnc	95.39	93.74	93.79	50.77	97.48
	CPC2	87.60	94.76	94.57	50.23	97.17
	LncFinder	94.00	**95.25**	**95.22**	**55.50**	/
*A*. *thaliana*	Prelnc2	**100.00**	**95.22**	**95.40**	**65.92**	**97.84**
	Prelnc	99.93	93.14	93.40	59.00	97.48
	CPC2	82.45	92.47	92.09	47.25	95.98
	LncFinder	99.48	90.61	90.95	51.83	/
*O*. *sativa*	Prelnc2	83.45	**97.82**	**96.10**	**69.40**	**96.64**
	Prelnc	**96.22**	82.06	83.02	46.70	96.09
	CPC2	78.17	77.27	77.33	31.66	83.63
	LncFinder	94.10	87.86	88.24	54.00	/
*Z*. *mays*	Prelnc2	**100.00**	**99.59**	**99.64**	**98.07**	**99.97**
	Prelnc	99.79	97.55	97.78	89.51	99.89
	CPC2	88.92	89.24	89.21	60.76	98.40
	LncFinder	98.55	92.33	92.97	73.45	/

^a^The numbers in bold are the highest parameters of the prediction results.

^b^ LncFinder gives a direct result rather than a probability value, so it cannot give the AUC.

### Validation of performance with various datasets

In addition to the comparative validation on the known dataset, we also performed model validation on the CPC2 dataset. To facilitate comparison, we directly apply the models trained in the previous section to the CPC2 dataset. The results (Table 5) demonstrate that our model still achieves good accuracy when using the trained model to predict the same data, especially for A. thaliana. We also find that our features are moderately reliable on the mouse dataset.

**Table 5:** Prediction results on CPC2 datasets.

Species	Methods	SEN%	SPE%	ACC%	MCC%
*Human*	Prelnc2	91.45	**100.00** ^a^	95.43	91.23
	Prelnc	**96.07**	98.89	**97.34**	**94.71**
	CPC2	92.69	96.00	94.18	88.39
	LncFinder	93.11	96.24	94.52	89.05
*Mice*	Prelnc2	95.81	90.15	92.70	84.71
	Prelnc	92.17	92.25	92.22	83.68
	CPC2	94.89	93.87	94.26	87.97
	LncFinder	**96.70**	**95.41**	**95.91**	**91.43**
*A*. *thaliana*	Prelnc2	**99.80**	99.89	**99.73**	98.36
	Prelnc	98.21	**100.00**	**99.73**	**98.94**
	CPC2	99.65	95.31	95.98	95.98
	LncFinder	96.72	93.44	93.95	80.77

^a^Numbers in bold represent the highest performance in each column.

### Generalization

#### Predict on NoCodeV5 datasets

We retrain and predict the NoCodeV5 data. The results are shown in Table 6. Our model achieves very good accuracy, on average much higher than the other three, suggesting that retraining on other datasets is better.

**Table 6:** Prediction on NONCODEv5.

Methods	*Human*	*Mouse*
Prelnc2	**97.42%** ^a^	**98.71%**
Prelnc	95.32%	96.32%
CPC2	93.95%	95.93%
LncFinder	94.26%	94.26%

^a^Numbers in bold represent the highest performance in each column.

#### Generalization on heterozygous datasets

When we assumed that there is no annotated data for training, we can only use the known model to predict the unknown data. Here, we employ the known model as the previous two sections to predict the heterozygous dataset and test its ACC on the unlabeled dataset, which contains 10 animal data and 9 plant data. The composition of the lncRNAs dataset is given in Table 7.

**Table 7:** Heterozygous dataset.

Animal		Plants	
Species	Total Number	Species	Total Number
*Human*	172,216	*B*. *napus*	8,212
*cow*	23,515	*Cucumber*	2,550
*Chicken*	12,850	*Soybean*	2,242
*Mouse*	131,697	*G*. *raimondii*	1,247
*C*. *Elegans*	3,154	*Apple*	1,843
*D*. *Melanogaster*	42,848	*P*. *trichocarpa*	2,248
*Zebrafish*	4,852	*Tomato*	3,822
*Yeast*	55	*Wheat*	12,427
*Platypus*	11,210	*Grape*	3,351
*Pig*	29,585		

**Fig 4:**
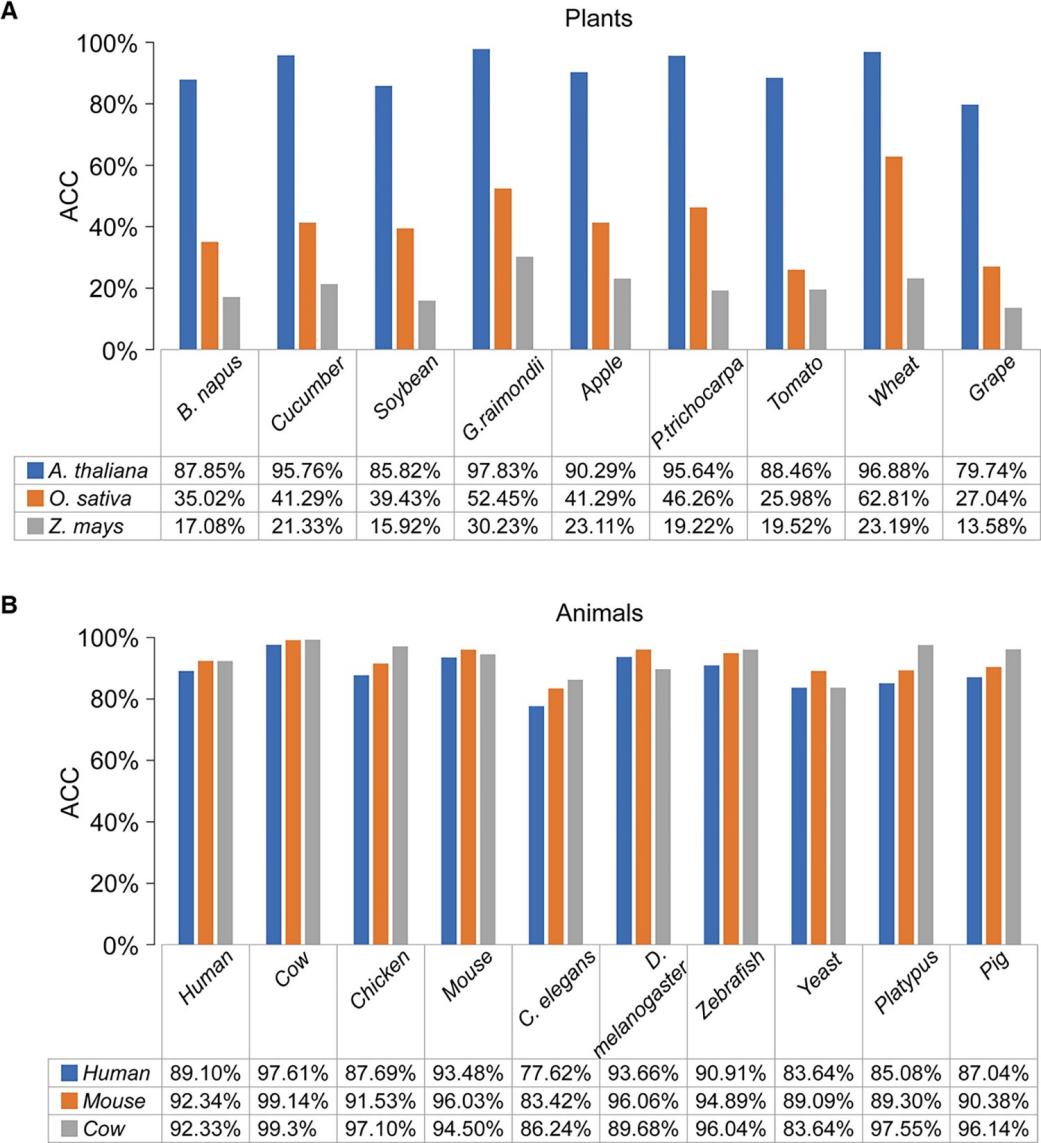
The result of generalization prediction using a known model. (**A**) The prediction results of the trained *Human*, *Mouse*, *Cow* models on other animal datasets, and on average the prediction results of *Mouse* are better. (**B**) The prediction results of the trained *A*. *thaliana*, *O*. *sativa*, *Z*. *mays* models in prediction of other plant datasets, on average *A*. *thaliana* prediction results are better.

We used the given models of *Human*, *Mouse*, and *Cow* to predict the animal data in turn, and then used the given model of *A*. *thaliana*, *O*. *sativa*, *Z*. *mays* to predict the plant data in turn (Fig 4A). The given model of animals has a good prediction ability for the data of unknown models, with an average of more than 93% in accuracy. However, plants present a strong difference, and the prediction results of the A. thaliana model, which is carefully studied, are outstanding, but the phenotypic ability of the other two models are poor (Fig 4B). Therefore, using known animal models to predict unknown animal models can be directly applied, with little impact on accuracy. However, plant models prefer those that are more fully studied and trained on more diverse and complete data.

## Discussion

As a modern signal analysis tool, the wavelet transform has some advantages in the frequency domain and in the time scale domain, which corresponds to the position scale. Therefore, some features of the sequence can be carefully explored. These features more or less depend on how effectively we can convert these biological sequences into numerical ones, so it is also important to choose a suitable transcoding method, which still needs to be investigated further. The wavelet feature of the biological sequence depends on the assumption that there is a higher protruding potential or protruding chemical properties in the sequence. Thus, we can use the difference in position to distinguish target positions or distinguish lncRNAs as described in this paper. In recent years, there are still some problems with this assumption.

First, the positions of transposons with higher hotspots or repeated segments are uncertain, so we need to deal with the failure of wavelet features caused by such positions or repetitions. Second, there are still some difficulties with the wavelet features of pseudo-proteins, and This is mainly due to that we cannot actually figure out how the proteins directly act on each other by the charge right.

On the one hand, unlike RNA or DNA sequences Who’s features can be directly calculated, the protein sequence is the result of three-coding translation, because we cannot determine the coding part of the RNA sequence or whether the fragment is directly involved in the coding, so that the wavelet transform of protein-coding can only transform the whole macroscopically, which is very time consuming and inaccurate. Currently, wavelet transform features have been used to predict target sites in some literature, so whether these annotated sites can be used in the identification of lncRNAs still requires additional research.

In Conclusion, in this work, we analyze the wavelet transform property of sequences and show that it is not only a strong classifier on plants, but also a highly discriminative classification property. After combining multiple existing sequence features, we employ a random forest classifier to classify the existing data, and the results demonstrate its high performance on metrics such as accuracy, especially on plant data. At the same time, we also find that wavelet transform features still have certain shortcomings, such as how to efficiently extract features from pseudo-proteins, time-consuming computations, and the hierarchical information of wavelet spectra cannot be fully employed. These are questions that need further investigation.

## Code access

The code in this article can be accessed online: https://github.com/spu112005/prelnc2.
